# Hybrid Metal Oxide/Biochar Materials for Wastewater Treatment Technology: A Review

**DOI:** 10.1021/acsomega.2c02909

**Published:** 2022-07-27

**Authors:** Ewelina Weidner, Elika Karbassiyazdi, Ali Altaee, Teofil Jesionowski, Filip Ciesielczyk

**Affiliations:** † Poznan University of Technology, Faculty of Chemical Technology, Institute of Chemical Technology and Engineering, Berdychowo 4, PL-60965 Poznan, Poland; ‡ University of Technology Sydney, School of Civil and Environmental Engineering, Centre of Green Technology, 15 Broadway, Ultimo NSW Sydney, New South Wales 2007, Australia

## Abstract

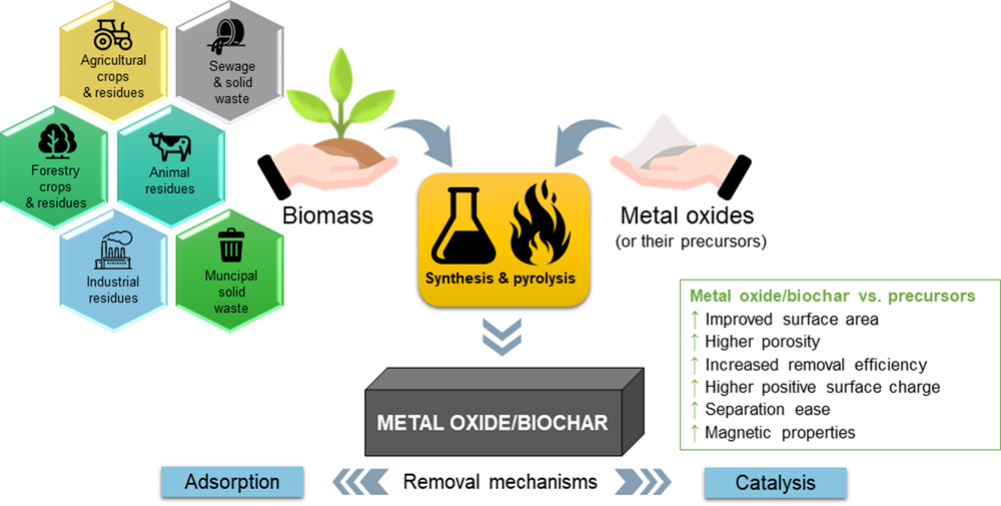

This paper discusses the properties of metal oxide/biochar systems for use in wastewater treatment. Titanium, zinc, and iron compounds are most often combined with biochar; therefore, combinations of their oxides with biochar are the focus of this review. The first part of this paper presents the most important information about biochar, including its advantages, disadvantages, and possible modification, emphasizing the incorporation of inorganic oxides into its structure. In the next four sections, systems of biochar combined with TiO_2_, ZnO, Fe_3_O_4_, and other metal oxides are discussed in detail. In the next to last section probable degradation mechanisms are discussed. Literature studies revealed that the dispersion of a metal oxide in a carbonaceous matrix causes the creation or enhancement of surface properties and catalytic or, in some cases, magnetic activity. Addition of metallic species into biochars increases their weight, facilitating their separation by enabling the sedimentation process and thus facilitating the recovery of the materials from the water medium after the purification process. Therefore, materials based on the combination of inorganic oxide and biochar reveal a wide range of possibilities for environmental applications in aquatic media purification.

## 1 Introduction

**Figure 1:**
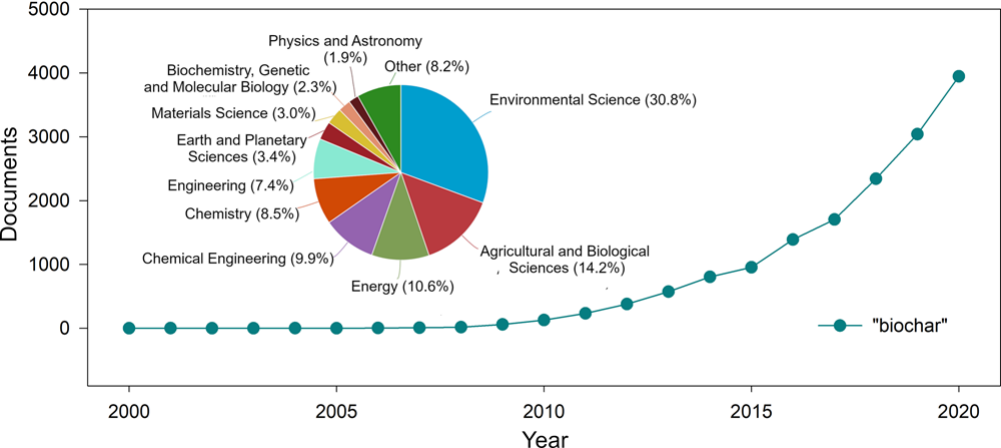
Graph of the number of documents about biochar versus the year of publication and pie chart showing the main domains in which those materials found application. The statistical data were obtained by searching the word “biochar” in the Scopus database in titles, keywords, and abstracts.

In line with sustainable development in mind, waste material management—biomass—is an inevitable obligation for science and industry.^1^ Biochar is a carbon-rich material produced in the thermal decomposition or pyrolysis of carbonaceous biomass in the absence, or under a limited amount, of oxygen.^2−4^ Basically, all carbonaceous organic matter can be used as a biochar precursor, including lignocellulose biomass, agricultural biomass (i.e., plant or animal biomass or manure), municipal and industrial residue, and activated sludge.^5,6^ According to Scopus, the first scientific article on biochar appeared in 2000, and the interest in this material has grown steadily over the past 20 years (Figure 1).

Currently biochar is used in many fields, but mostly in environmental sciences. Due to the low material cost and desirable properties such as high surface area, alkalinity, abundant oxygen-containing functional groups, and high cation exchange capacity,^7^ biochar has found application in wastewater treatment, as an adsorbent for the removal of contaminants such as nutrients, trace metals, pharmaceuticals, pesticides, dyes, metal(loids), volatile organic compounds, and polycyclic aromatic hydrocarbons.^3^ In comparison with polymeric and commercial adsorbents, biochar, due to its environmentally benign nature, low replacement cost, and practical application on a large scale, has attracted much attention in hazardous metal removal.^8^ Biochar owes its sorption properties to disordered valence sheets that generate incompletely saturated valences and unpaired electrons, which results in an increased number of active sites. A large amount of delocalized π electrons causes a negative charge of the biochar surface; thus, it behaves like a Lewis base, effectively attracting Lewis acids through processes of physi- and chemisorption.^4^ In addition, the presence of oxygen-containing and nitrogen-containing functional groups on the biochar surface enhances adsorption through acid/base interactions and hydrogen-bond formation.^4,9^ In addition to that, as biochar possesses carbon matrix, structural defect sites, and various surface functional groups, it is suitable for efficient use in photocatalytic reactions. Biochar has remarkable electrical conductivity, leading to its decreased electron/hole recombination rate during the photocatalytic process, thus enhancing the oxidation rate of the target compound.^10^ Moreover, it has been employed as an ideal support to disperse and mount active particles.^11^ All of these features make biochar an interesting alternative to activated carbon in the fields of adsorption and photocatalysis.

Despite its numerous advantages, biochar also has significant limitations. While pristine biochar reveals an excellent adsorption capacity for organic substances, it exhibits a very limited adsorption capacity for anionic pollutants.^12^ Moreover, raw biochar requires a long equilibrium time, due to its limited surface functional groups and porous structure.^13^ Additionally, the biomass source, reaction media, and processing conditions determine the biochar properties,^3,5^ which means that biochars will differ in the range of molecular structure and topology. The separation of biochar powders after the removal treatment causes significant difficulties, thereby entailing a secondary pollution problem.^14,15^ Therefore, numerous studies have been conducted to improve biochar properties, including chemical and physical approaches.^7^ To improve its properties for environmental applications, chemical processes such as acid and base modification, metal salt or oxidizing agent modification, and carbonaceous material modification are most often selected. Physical methods, mainly including steam and gas purging, have been less commonly used.^16^

## 2 Combination of Biochar with Inorganic Oxides

Incorporation of inorganic oxides into biochar is beneficial to its properties. Hybrid materials composed of biochar and metal oxides are never the sum or average of the properties of their components. Due to the connections formed between them, they show completely new, unique properties that reveal the advantages of both main elements.^17^ The dispersion of an inorganic oxide in a carbon matrix causes the creation or enhancement of surface properties and catalytic or magnetic activity and facilitates the recovery of the nanometer-sized materials.^18,19^ Unfortunately, due to its negative surface charge, biochar has a very low affinity for anionic impurities. Modification with positively charged metal oxides may change the surface properties and thus increase this affinity. Some metal oxides, such as TiO_2_ and ZnO, exhibit significant photocatalytic activity, and so their addition to biochar enhances its properties in this field. Moreover, the bulk density of biochar usually ranges from 80 to 320 kg/m^3^,^20^ depending on the difference in raw materials used as a biochar source and the particle size of the obtained biochar, translating into its packing degree. The low weight and small amounts, typically 1 g per liter of liquid, used for wastewater treatment make its further separation difficult. Addition of metallic species into biochars increases its weight, facilitating its separation by enabling the sedimentation process. Modification of biochar with magnetite (Fe_3_O_4_), which increases the hybrid’s magnetic properties, facilitates the separation process even more.

**Figure 2:**
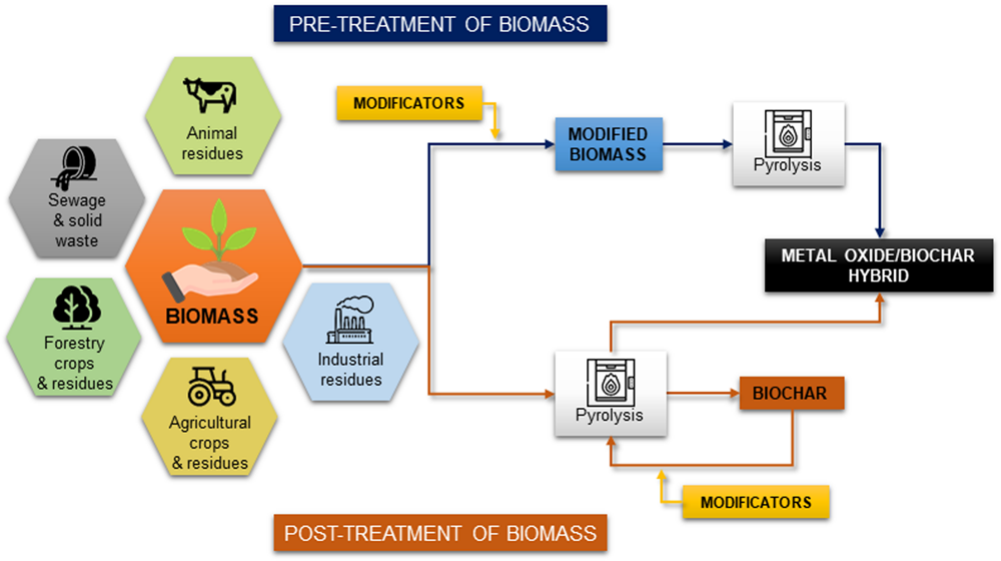
Schematic representation of the methods for fabrication of the metal oxide/biochar hybrid systems.

There are two equally frequently used methods to fabricate biochar-based metal oxide materials: (1) pretreatment of biomass by modifying the raw material used for the production of biochar by adding a metal oxide, or its precursor, and subjecting such a system to pyrolysis and (2) post-treatment of biochar with metal salts after the pyrolysis process.^16,17^ A schematic representation of these processes and the sources of biomass is given in Figure 2. Due to the lack of necessity for repyrolysis, the pretreatment approach is considered to be more energy efficient due to the simultaneous pyrolysis of biomass and the metal precursors.^21,22^ Moreover, the addition of the metal precursors before pyrolysis enables the occurrence of various reactions between them and the raw material, while modification after pyrolysis, if done at lower temperatures, does not initiate some additional reactions. Metals whose compounds are used to modify the biochar most often include titanium, zinc, and iron. During pyrolysis after addition of those metals to raw materials various scenarios can happen—in the case of titania, no reduction is observed, zinc can evaporate if pyrolysis is done at temperatures that are too high, and some reduction processes of iron can occur, resulting in the presence of oxide, metallic Fe, or Fe carbide, depending on the temperature. The introduction of various precursors of the same element into the system may result in different final properties. It should be remembered that often not all residues can be eliminated from the end material; thus, one should choose the precursor that will not weaken the desired properties—e.g. for a material with increased catalytic properties a sulfur-free precursor should be used, because sulfur is a known poison of many catalysts.

## 3 TiO_2_/Biochar Materials

TiO_2_ is probably the most thoroughly researched material used in catalysis and photocatalysis processes. Titania is also applied in areas that range from photovoltaics and photocatalysis to photoelectrochromics and sensors, as well as in antibacterial agents and nanopaints with a self-cleaning effect.^23^ It owes this position to a set of desirable features such as low price, nontoxicity, low environmental side effects, corrosion resistance, chemical stability, high oxidative potential,^24^ and most importantly, high catalytic activity.^25,26^ However, there is no rose without a thorn, and TiO_2_ has an equally substantial set of drawbacks. The greatest limitation of the practical application of TiO_2_ to a large extent is its wide energy gap (3.2 eV), which makes it only active on irradiation by UV light.^25,26^ Because the solar spectrum consists of only about 4% of UV light,^27^ pure TiO_2_ is not very effective under visible-light catalytic processes. Degradation of pollutants using UV radiation is burdened with additional costs related to the maintenance of the irradiation system. Improving the photocatalytic capacity of TiO_2_ in visible light would significantly reduce the cost of wastewater treatment. TiO_2_ also has a high electron/hole pair recombination rate in comparison to the rate of chemical interaction with the adsorbed species for redox reactions.^18,28,29^ Moreover, TiO_2_ particles exhibit a significant agglomeration tendency, making it hardly separable from the aqueous phase.^25^ In order to reduce the aforementioned imperfections of TiO_2_, it has been combined with other materials—by creating polyoxide systems and TiO_2_ deposition on a matrix. Carbon materials, such as activated carbon, carbon nanotubes, and even biochar are used in this role. Data collected from an analysis of articles devoted to the combination of TiO_2_ with biochar and its application in the wastewater treatment, are presented in Table 1.

**Table 1:** Data of Wastewater Treatment Processes using TiO_2_/Biochar Systems

material	feedstock	pyrolysis temp (°C)	surface area (m^2^/g)	pollution	initial pollution concentration (mg/dm^3^)	applied dose (g/dm^3^)	adsorption capacity (mg/g)	degradation method	removal eficiency (%)	ref
TiO_2_/biochar	hemp stem	500	17.4	ammonia nitrogen	100	0.03		catalysis (UV)	∼99.0	(30)
TiO_2_/CuO/biochar	hemp stem	500		ammonia nitrogen	100	0.03		catalysis (UV)	99.7	(30)
TiO_2_/biochar	*Daphnia magna*	325	383.0	sulfamethoxazole	10	5.00	2.2	adsorption + photocatalytic oxidation (UV)	91.0	(18)
TiO_2_/biochar	reed straw	300	102.2	sulfamethoxazole	10	1.25	6.6	adsorption + photocatalysis (UV)	91.3	(31)
Zn/TiO_2_/biochar	reed straw	500	169.2	sulfamethoxazole	10	1.25	8.0	adsorption + photocatalysis (Vis)	80.8	(25)
TiO_2_/biochar	paper sludge and wheat husks		26.3	Reactive Blue 69	20	1.50		sonocatalysis (UV)	98.1	(32)
TiO_2_/biochar	macroalgae	650		Methylene Blue	5	2.00	2.2	adsorption + photocatalysis (Vis)	99.0	(33)
TiO_2_/Fe/Fe_3_C/biochar	dewatered sewage sludge	800	50.3	Methylene Blue	200	1.00		adsorption + photodegradation (UV)	89.2	(19)
Fe_2_O_3_/TiO_2_/biochar	waste tea leaves	500	244.8	Methylene Blue	200	2.00		Fenton catalysis (Vis)	75.0	(11)
				Rhodamine B					60.0	
				Methyl Orange					40.0	
TiO_2_/biochar	walnut shells	500		Methyl Orange	20	0.25		photocatalytic oxidation (UV)	96.9	(34)
Ag/TiO_2_/biochar	walnut shells	700	35.2	Methyl Orange	20	0.25		catalysis (UV)	97.5	(26)
TiO_2_/biochar	*Salvinia molesta*	350	8.6	Acid Orange 7	20	0.10		photocatalysis (UV)	90.0	(35)
Fe/TiO_2_/biochar	rosin	800		Cr(VI)	150	0.80	77.2	adsorption	38.1	(36)
TiO_2_/biochar	raw corn cob	550	450.4	Cd(II)	50–300	1.00	72.6	adsorption	70.0	(37)
				As(V)			118.1			

Peng et al.,^30^ due to concern about the safety of water resources, researched an effective method to degrade ammonia nitrogen using materials based on hemp stem biochar and TiO_2_. Biomass material was soaked into an alcoholic solution of tetrabutyl orthotitanate and calcined. The best-performing samples were immersed into CuSO_4_ solution to obtain TiO_2_/CuO/biochar composites. The materials revealed excellent photocatalytic activity, attributed to the high surface area and the reduction of electron–hole pair recombinations as a result of the introduction of biochar and CuO. The TiO_2_/CuO/biochar catalyst revealed a high degradation rate of ammonia nitrogen of 99.7% under UV light and 60.7% under visible light. The catalysts remained stable after 10 cycles of degradation, retaining over 90% of ammonia nitrogen’s removal efficiency.

Hybrid materials made of titanium dioxide and biochar have also found application in water resource purification from sulfamethoxazole (SMX). Sulfamethoxazole is an antibiotic from the sulfonamide class, which is very effective in the treatment and prophylaxis of pneumonia. Due to its wide use in the treatment of human and animal diseases, high persistence in the environment, and inefficient degradation in the treatment plants,^38^ it is one of the most frequently detected antibiotics in surface water^39^ and groundwater.^40^ Emissions of high concentrations of SMX to the environment pose a threat to aquatic ecosystems, being toxic to aquatic organisms: i.e. fish and crustaceans.^41^ In 2016 Kim et al.^18^ prepared a TiO_2_/biochar composite by using an acid treatment of commercial biochar from *Daphnia magna* and a sol–gel method for TiO_2_ deposition onto the biochar surface. TiO_2_/biochar revealed higher adsorption capacity and higher mineralization rate of SMX under UV light in comparison to commercially available TiO_2_, due to the hydrophobic interaction between the biochar and SMX. The TiO_2_/biochar catalyst stayed stable after three cycles of photocatalysis, retaining a degradation efficiency on the level of 90–92%. The material obtained by Zhang et al.^31^ by using an analogous synthesis method but a different source of biochar—reed straw—had a similarly satisfactory SMX removal efficiency of 91% with UV irradiation. The same scientists^25^ improved the catalyst by inserting zinc particles into the TiO_2_/biochar hybrid. The Zn/TiO_2_/biochar material was able to degrade SMX from aqueous media without UV radiation and achieved an over 80% efficiency of photodegradation in visible light. In comparison with TiO_2_ and TiO_2_/biochar, Zn/TiO_2_/biochar had better photocatalytic activity under visible light due to zinc elements effectively inhibiting the agglomeration of TiO_2_ and hindering the combination of photogenerated electrons and holes.

Attempts were made to use systems based on TiO_2_ and biochar in order to remove organic dyes from the environment. From Table 1, it can be seen that TiO_2_/biochar materials were used by far for the degradation of Reactive Blue 69 (RB69), Methylene Blue (MB), Rhodamine B, Methyl Orange (MO), and Acid Orange 7 (AO7). Khataee et al.^32^ prepared a TiO_2_/biochar nanocomposite using the post-treatment of biochar by a sol–gel method with Ti(OBu)_4_ and used it in the process of sonocatalysis (ultrasonically assisted catalysis) of Reactive Blue 69, reaching a degradation efficiency of 97.5%. RB69 was first oxidized to aromatic intermediates and then to aliphatics and ultimately to H_2_O and CO_2_ by the attack of HO^•^ radicals. However, Fazal et al.^33^ created a composite based on macroalgae-derived biochar by depositing titanium(IV) isopropoxide on it by wet precipitation. In compariosn with the pristine components, the composite revealed higher charge separation, slower recombination of electron–hole pairs, and enhanced light absorption. A higher degradation efficiency of MB dye was also observed, 99%, while the pure biochar and TiO_2_ exhibited 85% and almost 43% efficiencies, respectively. Mian and Liu^19^ also synthesized a TiO_2_/Fe/Fe_3_C/biochar composite for Methylene Blue removal by a single-step route where sewage sludge and different ratios of nanoparticles (Fe and Ti) impregnated with chitosan were pyrolyzed at 800 °C. The obtained materials exhibited excellent MB degradation through a photoreaction and H_2_O_2_ activation, retaining their material stability, recyclability, easy separability, and low Fe-ion leaching after the catalytic processes. Meanwhile, Chen et al.^11^ developed an efficient Fenton catalyst to degrade three different dyes—Methylene Blue, Rhodamine B, and Methyl Orange. Fenton-like processes are widely applied for degradation of organic pollutants from aqueous solutions via highly active HO^•^ and O_2_^•–^ species. Biochar from waste tea leaves was soaked in a solution of Ti^4+^ and Fe^3+^ and pyrolyzed. Fe_2_O_3_/TiO_2_/biochar revealed high crystallinity and an irregular three-dimensional structure with abundant channels and holes. The porous structure properties of modified biochar were better than those of a pristine sample, exposing more active sites contributing to the reaction. Fe_2_O_3_/TiO_2_/biochar revealed significant reactivity for organic dye degradation due to the synergism between adsorption and oxidation. Lu et al.^34^ catalytically removed Methyl Orange from an aquatic environment. A TiO_2_/biochar composite was prepared using a direct hydrolysis method—the prepared walnut-shell-derived biochar was soaked in a TBOT solution and then calcined at 500 °C. Excellent MO removal at a level of 97% indicated that addition of the biochar to TiO_2_ could promote the photocatalytic properties. To increase the catalytic performance in their next study,^26^ the TiO_2_/biochar composite catalyst was modified with silver. The synergic connection of Ag, TiO_2_, and biochar resulted in an increased photocatalytic performance. Characterization tests indicated that Ag and TiO_2_ acted as electron donors and biochar acted as an electron acceptor, effectively promoting the separation of photogenerated electron–hole pairs. The Ag/TiO_2_/biochar composite exhibited MO degradation efficiency on the level of 97.48% and a high stability for up to five cycles. Silvestri et al.^35^ were interested in Acid Orange 7 removal using TiO_2_/biochar composites prepared using *Salvinia molesta* biochar as a carbonaceous anchor for TiO_2_. The influence of the synthesis method—sol–gel or mechanical mixing—and the type of titanium precursor—titanium isopropoxide (TTiP) or TiOSO_4_—on the properties of the obtained composites were investigated. For both synthesis routes, the composites prepared from TTiP showed a higher crystallinity, lower band gap, and larger pore size. The TiO_2_/biochar composite prepared by the mechanical mixing approach with the TTiP precursor resulted in the best removal performance, reaching 47% dye adsorption and 58% photocatalytic decolorization after 4 h.

Attempts were also made to use TiO_2_ composites with biochar to purify water from harmful metal ions. Yousaf et al.^36^ created an Fe/TiO_2_/biochar composite by soaking rosin-derived biochar and commercial TiO_2_ in a FeCl_3_ suspension and then pyrolyzing it at 1200 °C. The wet chemical coating process enabled the synthesis of Fe/TiO_2_/biochar as an efficient adsorbent for Cr(VI) ions, reaching a removal efficiency of ∼95% within the first 1 min of reaction. Simultaneous removal of cations and anions in wastewater was the subject of the studies of Luo et al.,^37^ who synthesized a TiO_2_/biochar material for cadmium and arsenic removal. A corncob-derived biochar was post-treated with butyl titanate by an ultrasonically assisted sol–gel route, resulting in an eco-friendly sorbent material. The Cd(II) and As(V) adsorption had a competitive effect in binary metal solutions, and the dominant adsorption mechanisms were ion exchange and complexation.

**Figure 3:**
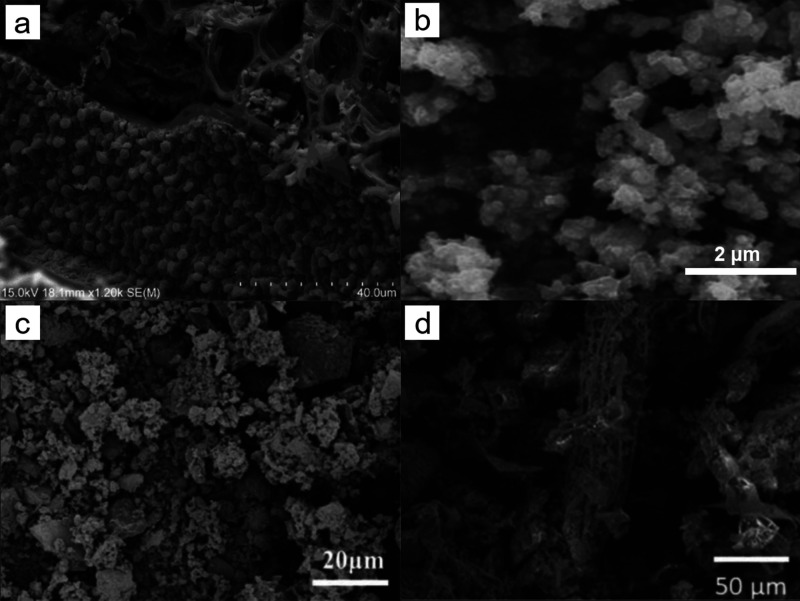
SEM images of titanium dioxide/biochar hybrids prepared by using the pretreatment methods (a) sol–gel (adapted with permission from ref (18)) and (b) wet precipitation (adapted with permission from ref (33)) and the post-treatment techniques (c) direct hydrolysis on biochar (adapted with permission from ref (34)) and (d) impregnation of biochar by mechanical mixing (adapted with permission from ref (35)).

The excellent catalytic performance of pristine TiO_2_ encouraged researchers to strengthen its weaknesses, such as a relatively low surface area and a large energy gap, through numerous modifications—including biochar incorporation. To summarize the data discussed above, the unwavering interest in TiO_2_/biochar composites in the field of degradation of pollutants in aquatic environments is noticeable. The addition of biochar increased the surface area of composite materials in comparison to pure TiO_2_ or biochar. The change in the surface properties and the morphology of TiO_2_/biochar hybrid materials is influenced by the synthesis method, the type of TiO_2_ precursor, and the source of the biomass. SEM images of titanium dioxide/biochar hybrid materials obtained by various methods are shown in Figure 3.

Hybrid TiO_2_/biochar materials have become very effective, usually reaching an efficiency of over 90%, in the degradation of pharmaceuticals, organic dyes, and other compounds. They fared slightly worse in eliminating harmful metal ions from the environment, achieving efficiency in the range of 30–70%. Either way, TiO_2_/biochar materials are an interesting alternative to traditional catalysts and adsorbents in wastewater treatment processes.

## 4 ZnO/Biochar Materials

Zinc oxide has a nontoxic nature,^42^ thermal stability,^43^ a porous micro-/nanostructure with high surface area, a good adsorption capacity,^44^ a wide band gap energy of 3.37 eV, and high electron mobility,^45,46^ as well asan exciton binding energy of ∼60 meV,^47^ which translates to excellent quantum efficiency and semiconductive properties.^48^ ZnO is recognized to be one of the most effective catalysts.^49^ This metal oxide is an effective and desirable adsorbent for anionic species from wastewater.^50^ The ZnO production technology is uncomplicated and economical, and substrates for the synthesis are relatively cheap. Zinc oxide can be produced by vapor deposition, precipitation in water solution, hydrothermal synthesis, a sol–gel process, precipitation from microemulsions, and mechanochemical processes.^51^ The preparation methods and synthesis conditions control its structure and morphology and thus its adsorption properties. ZnO also has biocidal and antibacterial properties, which are additional advantages in wastewater treatment processes. When considering of the above, versatile applications of ZnO in water purification processes through adsorption and catalysis are not surprising.

Therefore, the combination of unique properties of zinc oxide with biochar was the subject of research by many scientists.^17,52−59^ The hybrid systems they obtained were subjected to degradation tests of various compounds, including pharmaceuticals, harmful metal ions, etc. Details concerning the removal of impurities by composites based on biochar and zinc oxide collected through a literature study are presented in Table 2.

**Table 2:** Data of Wastewater Treatment Processes Using ZnO/Biochar Systems

material	feedstock	pyrolysis temp (°C)	surface area (m^2^/g)	pollution	initial pollution concentration (mg/dm^3^)	applied dose (g/dm^3^)	adsorption capacity (mg/g)	degradation method	removal eficiency (%)	ref
ZnO/biochar	crayfish shell	600	236.9	trichloroacetic acid	50	2.0	8.6	adsorption		(56)
ZnO/biochar	camphor tree leaves	650	915.0	ciprofloxacin	30–300	0.5	449.4	adsorption		(55)
ZnO/biochar	wheat husks and paper sludge	500	183.4	gemifloxacin	20	1.5		catalysis	83.7	(52)
ZnO/biochar	bamboo			Methylene Blue	20	2.0	0.823 mg/kg	adsorption + photocatalysis (UV)	83.9	(58)
ZnO/CMC/biochar	bamboo			Methylene Blue	20	2.0	17.01 g/kg	adsorption + photocatalysis (UV)	10.9	(58)
ZnO/biochar	*Corchorus capsularis*	700	62.2	Methylene Blue	20–100	0.5		adsorption + photoatalysis (UV)	99.0	(17)
ZnO/biochar	sewage sludge	450	15.5	Acid Orange 7	20	0.9	0.9	photo-oxidative process (persulfate oxidation)	93.8	(53)
ZnO/biochar	bamboo shoot shell	550	282.2	ReO_4_^–^	20–180	3.0	24.5	adsorption		(50)
ZnO/biochar	bamboo shoot shell	550	282.2	ReO_4_^–^	120–180	3.0	25.9	adsorption		(54)
ZnO/ZnS/biochar	corn stover (Zn contaminated)	600	397.4	Pb(II)	400	2.0	135.8	adsorption	35.1	(57)
				Cu(II)			91.2		39.0	
				Cr(VI)			24.5		21.3	
ZnO-betaine- biochar	commercial biochar		100.0	PO_4_^–^	10	3.0	265.5	adsorption	100.0	(59)

Long et al.^56^ conducted research on developing a ZnO/biochar composite for trichloroacetic acid (TCAA) removal. TCAA is carcinogenic and teratogenic and is a mutagenic byproduct of chlorine disinfection. A crayfish shell was immersed in zinc chloride and pyrolyzed at 600 °C. The obtained ZnO/biochar possessed a positively charged structure containing ZnO nanoparticles and a nearly 4 times higher surface area (236.93 m^2^/g) in comparison to the unmodified sample (63.79 m^2^/g). The presence of ZnO nanoparticles directly enlarged the surface area and strengthened the positive charge of the material. The material exhibited a high adsorption capacity for anionic TCAA. The process was probably based on surface adsorption and electrostatic attraction. Unfortunately, the TCAA adsorption was easily influenced by pH, coexisting anions, and temperature.

ZnO/biochar materials found application in wastewater treatment for the removal of pharmaceuticals. Ciprofloxacin (CIP) is considered to be one of the major antibiotic pollutants emitted from the treatment plants. Hu et al.^55^ used ZnO/biochar for the adsorption of ciprofloxacin. The biochar from camphor leaves, pretreated with ZnCl_2_ as a porogen, was doped with ZnO nanoparticles and calcined at 500 °C. The ZnO/biochar material revealed superior porosity in comparison to the pristine biochar, and the modification increased the surface area from 19 to 915 m^2^/g. The mechanism of CIP adsorption on ZnO/biochar is based on cation exchange, electrostatic interaction, and π–π stacking interaction. Another dangerous pharmaceutical pollutant is gemifloxacin (GMF), an antibiotic used in bacterial infections, known to cause severe pollution harm in aquatic environments. Gholami et al.^52^ synthesized a ZnO/biochar composite via a posttreatment method, where ZnO nanorods were grown on the biochar surface using a low-temperature hydrothermal procedure. ZnO nanorods were grown with a uniform size, high density, and random distribution on the porous structure of biochar. The ZnO/biochar nanocomposite showed a type IV isotherm with the wide hysteresis loop typical for mesoporous materials. The surface area increased from 68 m^2^/g for pristine biochar to 119 m^2^/g for the ZnO/biochar composite. Studies have revealed that functional groups on the surface of a material do not play a key role in degradation by advanced oxidation processes and in catalytic activity but they do increase the ZnO/biochar adsorption abilities. The presence of Zn–O stretching in ZnO/biochar increases its hydrophilicity while decreasing the affinity of the relatively hydrophobic GMF molecules to the catalyst surface.^60^ The synthesized material revealed a catalytic degradation of GMF on the level of almost 84%.

ZnO/biochar composites were successfully applied in the degradation of organic dyes. Scientists have mostly been interested in the photocatalytic abilities of these materials.^17,53,58^ Methylene Blue (MB) is used as a model compound in research directed on organic dye removal. The excessive discharge of this cationic dye in textile effluents was reported to be hazardous to the environment and human health.^61^ Sorption and degradation are commonly used techniques for organic dye removal; thus, Wang et al.^58^ conducted tests of the comprehensive removal of MB using ZnO/biochar composites encapsulated either with (ZnO/CMC/biochar) or with no (ZnO/biochar) sodium carboxymethyl cellulose (CMC). Composites were obtained via impregnation of bamboo-derived biochar with ZnCl_2_ and reduced by NaBH_4_ at 90 °C. CMC was used to manipulate the particle size and dispersion of ZnO on the carbonaceous surface. CMC’s presence contributed to the reduction of ZnO crystallite size but increased the band gap of ZnO/biochar, which may be ascribed to the disappearance of crystal defect vacancies appearing in the ZnO/biochar material. The addition of CMC to the structure increased MB sorption from 10.6% to 73.1% but decreased its degradation from 80.7% to 41.1%. Thus, the CMC could increase the electrostatic attraction between ZnO/biochar and MB. The compromised MB degradation may be caused by the reduced availability of hydroxyl and superoxide radicals and increased band gap energy of ZnO. Chen et al.^17^ used the photocatalytic abilities of a ZnO/biochar composite to remove Methylene Blue from aqueous media. Jute fibers, *Corchorus capsularis*, were pretreated with Zn(OAc)_2_ and carbonized at 700 °C. The activity of ZnO/biochar for MB photodecolorization was dominated by the morphology and ZnO content. The optimized Methylene Blue removal efficiency reached 99%, and the mineralization level was over 93% at pH 7.0 after 30 min of UV illumination. Kinetic studies indicated that both adsorption and photodegradation played an important role in the MB decolorization, but the surface photodegradation was the rate-controlling step. The recovered catalyst exhibited a high MB removal efficiency of over 80% during seven cycles.

Another approach to decrease organic dye pollution is an application of advanced oxidation processes (AOP) involving a persulfate (PS) oxidation, Fenton reaction, photocatalysis, and ozonation.^62,63^ Persulfate is a strong oxidizer able to generate sulfate radicals (SO_4_^•–^) through UV, heat, base, and carbon material and transition metal activation. Those radicals, together with H^+^ and HO^•^, are responsible for the process of Acid Orange 7 (AO7) decolorization. From that consideration, ZnO was previously used as a catalyst for photocatalysis and PS activation in the remediation of pollutants. The oxygen-containing functional groups on the surface of biochar may act as active sites of the electron-transfer mediator, which can result in the decomposition of persulfate. Guan et al.^53^ synthesized a composite of rectorite/sludge derived biochar supporting ZnO and evaluated its catalytic performance toward a heterogeneous photo-oxidative process (persulfate oxidation) for the degradation of Acid Orange 7 (AO7). Biochar was impregnated with zinc and calcined at 450 °C. ZnO particles, exhibiting significant crystal lattice planes, were well loaded on the amorphous biochar surface, resulting in a hybridized structure. The hybrid material had a higher surface area than pure ZnO (5.1 m^2^/g) but lower than that of pure sludge-derived biochar (17.6 m^2^/g), which showed that biochar was an excellent support material for ZnO loading. ZnO/biochar exhibited a high color removal ability (95%) and stable performance after three successive cycles. Hence, the material can activate PS for the remediation of wastewaters containing dyes or other organic contaminants.

Moreover, ZnO/biochar composites turn out to be effective in a purifying treatment of wastewater from harmful metals. Hu et al.^50^ prepared nano-ZnO functionalized biochar in order to use it in selective rhenium adsorption. Nitric acid functionalized biochar was mixed with zinc acetate dehydrate (ZnO precursor) dissolved in ethanol. The mixture underwent a solvothermal treatment and nitrogen pyrolysis to give a zinc/biochar composite. This method allowed the creation of highly dispersed ZnO nanoparticles bound to a biochar matrix with superhydrophobicity to give a water contact angle of about 151–156°. Due to the synergistic effect of surface superhydrophobicity and metal affinity, ZnO/biochar material revealed excellent selectivity and high efficiency to Re(VII), even in the presence of various competing ions, reaching a maximum Re(VII) adsorption capacity of 24.5 mg/g in selective tests. The adsorption mechanisms revealed that the inner-sphere complexation on a homogeneous surface is the dominant interaction, while liquid film diffusion was considered to be the rate-controlling step. In 2020, researchers conducted a model removal study on the same material using ReO_4_^–^ as a surrogate for radioactive pertechnetate (TcO_4_^–^), providing a feasible pathway for scale-up to produce highly efficient and cost-effective biosorbents for the removal of radionuclides.^54^ Li et al.^57^ synthesized nano-ZnO/ZnS-modified biochar via a low pyrolysis of the contaminated corn stover obtained from a biosorption process. The resulting material exhibited a rougher structure and a much higher surface area (*S*_BET_ = 397.4 m^2^/g) in comparison to pristine biochar (*S*_BET_ = 102.9 m^2^/g). The inserted zinc mineral was evenly anchored on the biochar surface as nano-ZnO/ZnS. Due to the presence of hydroxyl groups on the surface of nano-ZnO/ZnS particles and the well-developed porous structure catalyzed by the zinc salt during the pyrolysis process, the obtained hybrid revealed strong sorption affinity toward Pb(II), Cu(II), and Cr(VI), resulting in better adsorption performance in comparison to common biochar in metal removal. Nakarmi et al.^59^ conducted research on ZnO/betanine/biochar for removal of phosphate ions—one of the most costly and complex environmental pollutants, whose presence decreases water quality and limits access to clean water. Commercial biochar was impregnated with nano-ZnO in the presence of glycine betaine. Morphology studies exhibited the presence of spherical ZnO nanoparticles on the surface of biochar. An FT-IR analysis of the obtained composite showed connections between betaine and the biochar’s surface. No direct bonds between ZnO and the surface of the biochar were recorded; hence, the role of a binder was attributed to betanine. Despite having the lowest surface area of the discussed ZnO/biochar hybrids (see Table 2), the obtained material exhibited high phosphate removal efficiency—100% after 15 min in a 10 mg/dm^3^ phosphate solution. Tests with real wastewater solutions also gave positive results. It has been shown that the pH and the presence of coexisting ions in the aqueous solutions do not affect the adsorbent’s performance, proving it to be a useful alternative in phosphate ion removal.

**Figure 4:**
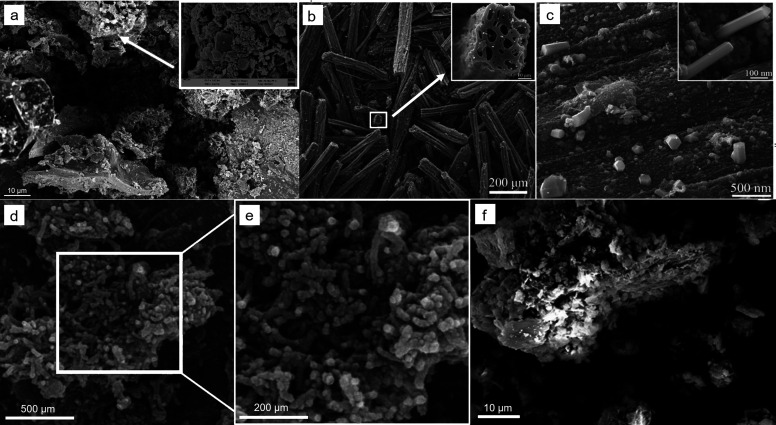
SEM images of zinc oxide/biochar hybrids prepared by using the pretreatment method (a) pyrolysis of biomass impregnated with zinc chloride (adapted with permission from ref (56)), (b, c) thermolysis of zinc acetate impregnated biomass (adapted with permission from ref (17)), (d, e) impregnation of biochar by mechanical mixing (adapted with permission from ref (52)), and (f) post-treatment impregnation with zinc nitrate (adapted with permission from ref (53)).

To summarize the data discussed above, it is clear that materials based on ZnO and biochar have numerous applications in water and wastewater treatment from various pollutants, showing a significant affinity for pharmaceuticals, organic dyes, and harmful metal ions. ZnO/biochar materials were synthesized by both pretreatment of biomass with zinc salts and impregnation of the fabricated biochar with zinc compounds, resulting mostly in obtaining irregular ZnO particles loaded on the surface of biochar, which exhibits a favorable decontamination capacity for wastewater treatment. The differences in the structure and morphology of the obtained ZnO/biochar materials are illustrated in Figure 4, showing the hybrids obtained using various synthesis methods, zinc oxide precursors, and biomass sources. The surface area of the resulting materials, ranging from 62 to 915 m^2^/g, was dependent on the pyrolysis temperature and feedstock used for biochar fabrication. The ZnO/biochar hybrids revealed a potential for the removal of diversified pollutants, which makes them universal materials for aqueous media purification and can be beneficial for treatment of sewage containing a large amount of slightly recognized pollutants.

## 5 Fe_3_O_4_/Biochar Materials

Magnetite, Fe_3_O_4_, is a widespread iron oxide exhibiting magnetic properties. It is one of the best-known and widely applied iron oxides. It has been used, among others, in ultrahigh-density magnetic storage media,^64^ ferrofluids,^65^ and biomedical applications such as MRI contrast enhancement, tissue repair, and drug delivery.^66^ Fe_3_O_4_ is made up of both ferrous (Fe^2+^) and ferric (Fe^3+^) ions—as a result, its synthesis and growth are possible only in an environment where oxidized and reduced states of iron are present and maintained.^67^ This oxide has a cubic inverse spinel structure, where oxygen forms a face-centered-cubic packing and iron cations occupy the interstitial tetrahedral and octahedral sites.^68^ Electrons are able to change their positions between Fe^2+^ and Fe^3+^ ions at room temperature,^69^ enabling the phenomenon of superparamagnetism. Superparamagnetism appears when the electrons in the atomic orbital are arranged in an orientation to generate magnetism under the influence of an external magnetic field. When the magnetic field disappears, the electrons return to their original orientation, causing a lack of magnetic properties.^70^ In addition to its magnetic properties, Fe_3_O_4_ exhibits features desired in wastewater treatment processes, such as abundance,^71^ biocompatibility,^72^ eco-friendly nature,^73,74^ and high reactivity.^71^ Magnetite has a positive surface charge at a pH of lower than 6.5,^75^ facilitating the effective adsorption of negatively charged pollutants by attractive electrostatic interactions at a pH of below 6.5.^76^

In terms of removal of pollutants from aquatic systems, the addition of magnetite is mainly dictated by the desire to impart magnetic properties to the adsorbents or catalysts, significantly facilitating their separation after the removal process. Materials with magnetic abilities can be easily removed by applying a magnetic field, while no fouling issue occurs with filtration systems^77,78^ and no secondary pollution is caused.^71,79^ Magnetic field separation is also economically advantageous.^79^ As it is known that steel companies produce enormous amounts of metal waste mainly composed of Fe_3_O_4_ nanoparticles, the recovery and reuse of waste magnetite in the purification of aquatic environments would ensure a promising source for iron and let the industry take a step forward in terms of waste-free production.^80^

Thus, research in the field of inducing magnetic properties into biochar was conducted.^81,82^ Various methods to synthesize magnetic carbonaceous materials have been presented in the literature, including the chemical coprecipitation of Fe^3+^/Fe^2+^ on biochar,^83,84^ presaturation of biomass in an iron precursor (such as Fe(NO_3_)_3_ or FeCl_3_) followed by pyrolysis,^38,85−88^ hydrolysis of the iron salt Fe(NO_3_)_3_ onto biochar,^89^ mechanical mixing of magnetic particles and biochar,^90,91^ a solvothermal method,^92^ and an electromagnetization technique.^93^ However, coprecipitation is the most popular method; it may reduce or cover adsorbent pores, causing change or inactivation of some adsorption sites.^94^ By using hydrolysis and pyrolysis, the magnetic properties of the Fe_3_O_4_/biochar composite may be reduced.^95^ Additionally, pyrolysis requires providing energy and can result in an uneven iron distribution.^77^ The solvothermal approach requires expensive autoclaves and makes observation of the reaction process impossible.^96^ Various iron compounds impart magnetic properties to materials; however, this work focuses on those in which only pure magnetite was present or it was the vast majority of iron compounds.

Hybridizing Fe_3_O_4_ with biochar provides not only its magnetization but also other benefits such as creating a large number of hydroxyl groups onto the biochar surface^71^ and improving the visible light sensitivity.^79^ Since biochar acts as an effective support, the aggregation of magnetite on Fe_3_O_4_/biochar materials is inhibited.^71^ Moreover, due to the positive surface charge of magnetite, it is expected that after modification of biochar by Fe_3_O_4_, the attractive electrostatic interactions at pH < 6.5 will also increase.^90^ These properties have made Fe_3_O_4_/biochar highly useful in the development of novel separation processes. Detailed data regarding the use of Fe_3_O_4_/biochar composite materials in wastewater treatment are presented in Table 3.

**Table 3:** Data of Wastewater Treatment Processes Using Fe_3_O_4_/Biochar Systems

material	feedstock	pyrolysis temp (°C)	surface area (m^2^/g)	pollution	initial pollution concentration (mg/dm^3^)	applied dose (g/dm^3^)	adsorption capacity (mg/g)	degradation method	removal eficiency (%)	ref
Fe_3_O_4_/biochar	watermelon rinds	500	111.2	Tl(I)	20	0.5	1123.0	adsorption + catalytic oxidation	99	(97)
Fe_3_O_4_/biochar	rice husk	500	109.0	U(IV)	0.04 mol/dm^3^	0.4	53.2	adsorption	96.8	(8)
				Pb(II)			110.0		91.7	
Fe_3_O_4_/biochar	wheat stalk	600	31.0	Pb(II)	100	1.0	179.9	adsorption		(98)
	rice husk		224.0				73.3			
Fe_3_O_4_/biochar	aerobic granular sludge	200		Pb(II)	15	0.3	37.9	adsorption	90	(99)
NH_2_/Fe_3_O_4_/biochar							46.6			
Fe_3_O_4_/biochar	crab shell	500	74.5	Pb(II)	50	1.0	62.4	adsorption	86	(100)
				As(III)	20		15.8		93	
Fe_3_O_4_/biochar	waste green wood	800–1000	320.1	As(III)	10	2.0	5.5	adsorption	68	(101)
Fe_3_O_4_/biochar	*Guadua chacoensis* culms	700	28.9	As(V)	10	2.0	90.0	adsorption	∼100.0	(102)
Fe_3_O_4_-KOH/biochar			482.4				85.0			
Fe_3_O_4_/biochar	*Phragmites australis*	600	232.7	Sb(V)	50	1.0	2.0	adsorption		(103)
Ce/Fe_3_O_4_/biochar^PC^			230.7				24.8			
Ce/Fe_3_O_4_/biochar^ST^			269.9				8.5			
Fe_3_O_4_/biochar	phoenix tree leaves	500	83.6	Cr(VI)	100	2.0	30.8	adsorption	98.2	(92)
Fe_3_O_4_/biochar	commercial biochar (wood)	900	312.6	PO_4_^3–^	25	0.4	82.5	adsorption	∼100	(104)
Fe_3_O_4_/biochar	*Pinus radiata* sawdust	650	125.8	sulfamethoxazole	21	2.0	13.8	adsorption		(38)
Fe_3_O_4_/biochar	hickory chips	600	90.6	Methylene Blue	100	0.2	500.5	adsorption	90.1	(90)
Fe_3_O_4_/biochar	brown marine macroalgae	600	337.0	Acid Orange 7	50	1.0	297.0	adsorption		(93)
Fe_3_O_4_/biochar	*Calotropis gigantea* fiber	600		PFOA	50	0.4	131.4	adsorption	100.0	(105)
				PFOS			136.5			
Fe_3_O_4_/FeOH·4H_2_O/biochar	sawdust	600	120.7	PFOS	0.5–325	0.7	194.6	adsorption		(106)
Fe_3_O_4_/biochar	commercial biochar (wood)	900	312.6	crude oil	spill: 2 g/25 mL	1.0	3.3	adsorption	>90	(107)
LA/Fe_3_O_4_/biochar			37.3				5.7			
Fe_3_O_4_/LA/biochar			30.6				6.2			

From the data collected in Table 3, it is visible that Fe_3_O_4_/biochar composites are mostly used in the field of harmful metal ion removal, including heavy metals such as thallium,^97^ uranium,^8^ and lead.^8,98−100^ Thallium, similarly to lead, is listed as a priority pollutant in many countries^108^ due to its extreme toxicity to the environment and living organisms. Thallium on the first oxidation level—Tl(I)—is the dominant form in the aquatic environment,^109^ where it is extremely mobile and persistent. Moreover, it is very heavily sorbable on traditional adsorbents.^110^ Paying attention to the often-overlooked environmental pollution phenomenon with thallium, Li et al.^97^ developed a Fe_3_O_4_/biochar adsorbent showing a very high affinity for Tl(I) ions with an adsorption capacity of 1123 mg/g. Watermelon-derived biochar was post-treated with dissolved FeCl_3_·6H_2_O and FeSO_4_·7H_2_O. The obtained material was characterized by a rugged and porous structure with irregular pores and holes. The Fe_3_O_4_/biochar surface area was higher than that of pristine biochar (14.1 m^2^/g), which implies that modification with magnetite has a significant influence on the structure and morphology, increasing the adsorption abilities. Also, such a combination was beneficial for the magnetic properties—the saturation magnetization of Fe_3_O_4_/biochar was equal to 31.54 emu/g, while for pure magnetite it was −22.68 emu/g. Precipitation of Tl_2_O_3_ on the surface of a porous Fe_3_O_4_/biochar composite, caused by oxidation and complexation of Tl(I) ions with surface hydroxyl groups, was thought to be the main mechanism of Tl(I) removal. The magnetite/biochar adsorbent had a fast oxidation rate, high adsorption capacity, and facile separability and was efficiently regenerated by HNO_3_ treatment, making the proposed removal route a promising method for decreasing thallium pollution. Uranium and lead contamination is a particular problem in highly developed countries, such as China.^8^ According to the World Health Organization (WHO) guidelines, the maximum allowable concentration of uranium and lead in drinking water should be lower than 10 and 15 μg/dm^3^, respectively. Therefore, Wang et al.^8^ used Fe_3_O_4_/biochar material, obtained via mechanical mixing of rice-husk-derived biochar with hydrothermally synthesized magnetite particles, for U and Pb elimination. After magnetic modification, the porosity, surface area, hydrophobicity, and reusability of material were effectively improved by approximately 1–2 times. Adsorption mechanisms of Pb(II) and U(VI) on the surface of Fe_3_O_4_/biochar were found to be electrostatic interaction and surface complexation. The adsorption of lead was mainly via physisorption, while uranium was mostly chemisorbed. Lead adsorption on Fe_3_O_4_/biochar was also investigated by Li et al.,^98^ who prepared wheat-stalk- and rice-husk-derived biochars and physically comixed them with commercial Fe_3_O_4_. The rice-husk-derived Fe_3_O_4_/biochar composite had much higher surface area in comparison to that based on wheat stalk biochar. Both of them revealed similar saturation magnetizations of 26.1 (rice husk) and 28.6 emu/g (wheat stalk), close to the results obtained by previously mentioned composites prepared by Li et al.,^97^ but were much lower than that of pure Fe_3_O_4_ (61.0 emu/g). The best adsorption capacity was noted for the wheat-stalk-derived sample, which had the lowest surface area, confirming that the surface area has a very weak correlation with Pb^2+^ adsorption.^111,112^ Adsorption mechanisms onto Fe_3_O_4_/biochar surface include conjugation adsorption, ion exchange, and Fe–O coordination as well as reactions of coprecipitation and complexation. While the proposed fabrication method seems to be a promising one to design eco-friendly magnetic chars with excellent adsorption capacity for water treatment, adsorbent reusability tests were not conducted. Huang et al.^99^ additionally modified magnetite/biochar with aminopropyltriethoxysilane (APTES) in order to increase the affinity of hazardous metal ions due to the strong metal chelation of the amino group. Solvothermally synthesized Fe_3_O_4_ and aerobic granular sludge/biochar were mixed with APTES in an aquatic environment. Epichlorohydrin, urea, and NaOH were used for the cross-linking process. Modification with amine contributed to a slight increase in the experimental sorption capacity in relation to Pb^2+^ ions—–from 37.9 to 46.6 mg/g. The adsorption mechanism seems to be based on surface complexation, electrostatic attraction, and precipitation phenomena. The adsorbent was stable after five adsorption–desorption cyclesa maintaining a lead adsorption efficiency of 88%. Chen et al.^100^ synthesized an adsorbent for the simultaneous removal of lead and arsenic ions by loading Fe_3_O_4_ nanoparticles on calcium-rich biochar derived from crab shells. Calcium is believed to exchange with lead and reduce its availability.^113^ Crab-shell-derived biochar revealed higher adsorption capacity for lead in comparison to aerobic-granular-sludge-derived Fe_3_O_4_/biochar (even that functionalized with amine) but lower in comparison to Fe_3_O_4_/biochar materials obtained from rice husk and wheat stalk. The materials revealed a satisfactory synergic effect on lead and arsenic adsorption—the As(III) addition enhanced Pb(II) removal by 5.4–18.8%, while the presence of Pb(II) suppressed As(III) removal by 5.8–17.8%. As has been mentioned before, arsenic pollution is recognized as one of the world’s greatest environmental hazards,^114^ because its inorganic form is strongly carcinogenic and highly toxic. Navarathna et al.^101^ synthesized a magnetic composite by the deposition of magnetite from a FeCl_3_ and Fe_2_(SO_4_)_3_·7H_2_O solution on the surface of commercial biochar. Researchers conducted batch adsorption tests on the real wastewater solutions originating from industry in Seattle, WA, containing As(III) (4 ppm in final the solution), resulting in lowering the arsenic content to below the WHO tolerance limit of 0.2 mg/L. During the adsorption of As(III) onto the Fe_3_O_4_ surface, a portion of As(III) was converted to less toxic As(V). This phenomenon pushed researchers to plan an effective way to eliminate As(V) in their further research,^102^ in which they used *Guadua chacoensis* derived biochar and investigated the influence of the KOH activation on the adsorption process. At naturally occurring aqueous arsenate concentrations, Fe_3_O_4_/biochar achieved removal efficiency of 100% (*q*_e_ = 5 mg/g at 25 °C). A robust adsorption performance in the presence of competing ions in the model and real arsenate wastewaters was observed and was not significantly affected by pH in the range 5–9. The proposed sorption mechanism is iron leaching, followed by precipitation of iron arsenate insoluble products onto the Fe_3_O_4_/biochar surface. KOH modification did not improve the adsorption capacity in relation to As(V) ions and even worsened it slightly. Apart from arsenic, another metalloid that poses a significant threat to the environment is antimony. Despite being 10 times less toxic than Sb(III), Sb(V) shows much greater mobility, stability, and solubility in polluted wastewater.^115^ Therefore, the subject of research conducted by Wang et al.^103^ was the removal of Sb(V) using magnetite/biochar composites and cerium-modified magnetite/biochar composites. Enrichment of the adsorbent with cerium results in a significant improvement in the adsorption properties toward anionic pollutants, i.e. arsenic;^116−118^ as arsenic and antimony are rare-earth metals that have a similar behavior, it was hypothesized that the modification with cerium would improve the sorption properties of the material toward antimony. Biochar was obtained from pyrolysis of *Phragmites australis* at 600 °C. The cerium-doped magnetic adsorbents were synthesized using chemical coprecipitation (Ce/Fe_3_O_4_/biochar^PC^) and solvothermal methods (Ce/Fe_3_O_4_/biochar^ST^). In terms of obtaining the highest sorption capacity, coprecipitation method was superior to the solvothermal method, and Ce oxide was the main contributor to the enhancement in Sb(V) adsorption. While the magnetic performance decreased after Ce doping, the material retained a satisfactory separation ability. Mechanisms controlling Sb(V) adsorption on Ce/Fe_3_O_4_/biochar^PC^ involved an inner-sphere surface complexation, hydrogen bonding, electrostatic attraction, and ligand exchange. Attempts were also made to adsorb chromium ions on the surface of Fe_3_O_4_/biochar. Liang et al.^92^ used a one-pot solvothermal method to obtain a magnetite/biochar composite using biochar derived from phoenix tree leaves as the carbonaceous matrix. The obtained material revealed a high affinity for Cr(V) ions, reaching an adsorption capacity of 55.0 mg/g. A study of the mechanism revealed that biochar provided binding sites for Cr(VI) and electron-donor groups for the reduction of Cr(VI) to Cr(III), while Fe_3_O_4_ nanoparticles were mainly involved in the immobilization of Cr(III) through the formation of Fe(III)–Cr(III) hydroxide. Fe_3_O_4_/biochar was found to be effective for chromium removal and remained satisfactorily stable after seven cycles, retaining 84% efficiency.

Karunanayake et al.^104^ were interested in phosphate removal by means of adsorption onto Fe_3_O_4_/biochar. Cheap commercial biochar with a high surface area (695 m^2^/g) was modified by chemical coprecipitation of Fe_3_O_4_ from Fe^3+^/Fe^2+^ aqueous NaOH. Fe_3_O_4_/biochar removed ∼90.0 mg/g of phosphate from water, reaching an approximately 20 times higher value of the capacity reported for neat magnetite particles (∼5.1 mg/g).

As mentioned in TiO_2_/Biochar Materials, sulfamethoxazole pollution is a significant environmental problem. Reguyal et al.^38^ applying oxidative hydrolysis of FeCl2, obtained single-phase Fe_3_O_4_ nanoparticles formed on the surface of biochar from *Pinus radiata* sawdust and used it as an SMX adsorbent. The adsorption mechanism study showed that SMX has almost no sorption affinity for the Fe_3_O_4_; thus, the material was limited to the biochar adsorption ability—it occurs through attachment of the SMX methyl group to the hydrophobic surface of biochar. The presence of Fe_3_O_4_ on the biochar matrix reduced the surface area and SMX adsorption capacity but enabled an easy magnetic separation after the treatment process.

With regard to the use of Fe_3_O_4_/biochar systems in the removal of organic dyes from aquatic environments, attempts were made to use them to eliminate Methylene Blue and Acid Orange 7. Li et al.^90^ proposed a solvent-free synthesis of magnetic hickory-chip-derived biochar through ball-mill extrusion with Fe_3_O_4_ nanoparticles and applied it for MB adsorption. The high MB adsorption capacity (500.5 mg/g) of the Fe_3_O_4_/biochar was attributed to the increased surface area, open pore structure, functional groups, and aromatic carbon–carbon bonds (promoting π–π and electrostatic interactions). An analogously prepared material, but with activated carbon as a carbonaceous matrix, revealed a lower MB adsorption capacity of 304.2 mg/g. The Fe_3_O_4_/biochar adsorbent was easily separated magnetically and revealed good reusability, maintaining ∼80% of its removal capacity after five adsorption–desorption cycles. Ball-mill mixing of biochar and magnetite is a cheap and eco-friendly method to create effective, low-cost magnetic adsorbents for dye contaminant removal. Jung et al.^93^ prepared a Fe_3_O_4_/biochar composite via an electromagnetization technique of brown marine macroalgae for Acid Orange 7 removal. The material was prepared by a stainless steel electrode based electrochemical system and then subjected to pyrolysis in 600 °C. A physicochemical analysis of the obtained material revealed that magnetite was embedded in the biochar. Fe_3_O_4_/biochar revealed satisfactory adsorption properties for AO7, with a fine porosity and a surface area of 337 m^2^/g.

Perfluoroalkyl and polyfluoroalkyl substances (PFASs) are a large group of synthetic organofluoride compounds possessing a number of unique properties such as high surface activity, water repellency, acid–base resistance, and chemical stability, thanks to which they have been widely used in polymer, surfactant, pesticide, and food packaging industries since the 1940s. Their huge consumption has caused significant emissions to the environment, where they are easily bioaccumulated and reveal eco-toxicological effects. Perfluorooctanoic acid (PFOA) and perfluorooctanesulfonate (PFOS) were the most extensively produced and studied of PFASs. Their persistence in the environment is related to the effect of an aggregate of strong carbon–fluorine bonds (485 kJ/mol).^119^ Therefore, finding a solution that allows for their effective elimination from the environment is very important. Niu et al.^105^ investigated the adsorption performance of the Fe_3_O_4_/biochar composite for PFAS pollutants. The synthesis consisted of mechanical mixing of *Calotropis gigantea* fiber derived biochar with Fe_3_O_4_ nanoparticles obtained from FeSO_4_·7H_2_O, polyvinylpyrrolidone and NaOH and carbonization in 400 °C. With the loading of Fe_3_O_4_ nanoparticles and secondary pyrolysis, the resulting Fe_3_O_4_/biochar showed a shortened, roughened, and partially unclosed tubular structure in comparison to untreated biochar. The obtained material reached an adsorption equilibrium after 1 h for PFOA and 2 h for PFOS, attaining adsorption capacities of 136.5 mg/g (PFOA) and 131.4 mg/g (PFOS). The coexisting ions had a beneficial influence on the adsorption efficiency, in particular for multivalent metal cations. The driving force of fast adsorption of PFAS was hydrophobic interactions. The obtained adsorbent was easily regenerated and recycled six times and maintained an efficiency of above 50%. Hassan et al.^106^ were interested in developing an efficient adsorbent for PFOS using waste materials—sawdust and raw red mud. Therefore, they obtained a material including biochar, magnetite, ferrihydrite, and desilication products that reached a high adsorption capacity of 194 mg/g. Similarly to the work of Niu et al.,^105^ hydrophobic and electrostatic interactions were essential mechanisms of PFOS adsorption. Kinetic studies confirmed the occurrence of both physisorption (diffusion and hydrophobic interaction) and chemisorption (electrostatic interaction and ion exchange) of PFOS onto the adsorbent. Combining waste material management with their subsequent use brings a great benefit in the field of environmental purification.

An important issue from the point of view of environmental protection was taken up by Navarathna et al.,^107^ who used Fe_3_O_4_/biochar and lauric acid modified Fe_3_O_4_/biochar for oil spill removal. Their adsorbents were prepared through Fe_3_O_4_ precipitation from FeCl_3_ and FeSO_4_ solution on the commercial biochar’s surface. Modification with lauric acid gave the adsorbent a floating ability and increased the oil adsorption capacity. Two modification approaches were made: coating Fe_3_O_4_/biochar with lauric acid (LA/Fe_3_O_4_/biochar) and coating lauric acid/biochar with Fe_3_O_4_ (Fe_3_O_4_/LA/biochar). All tested materials rapidly (≤15 min) took up significant amounts (up to 11 g oil/g of adsorbent) of four (engine, transmission, machine, and crude) oils from fresh and simulated seawater. The adsorbent was easily magnetically separated, recycled a few times, and after exhaustion combusted to produce useful heat while avoiding toxic or undesirable waste disposal.

**Figure 5:**
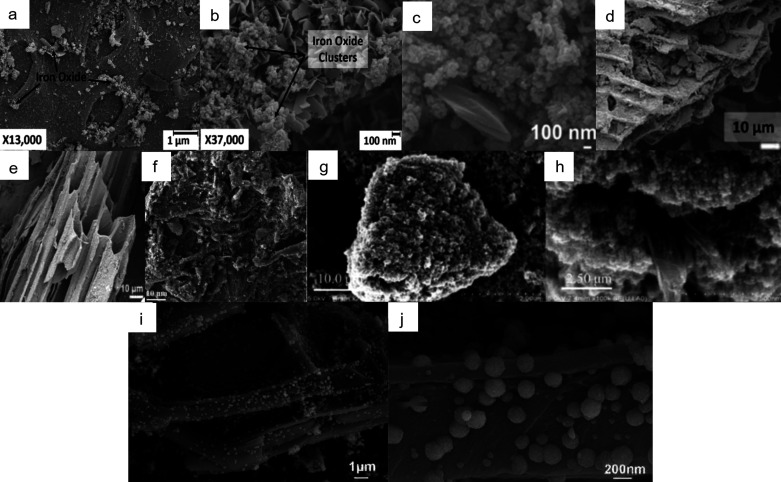
SEM images of magnetite/biochar hybrids prepared by using different approaches: (a–h) post-treatment of biochar by Fe^2+^ and Fe^3+^ coprecipitation (adapted with permission from refs (102, 100, 104, 97, 107, and 101)) and (i, j) one-pot solvothermal synthesis (adapted with permission from ref (92)).

The overall literature study indicates that Fe_3_O_4_/biochar materials reveal properties desired in adsorption processes of pollutants varying in structure and behavior. Magnetite/biochar materials have been extensively studied in the case of removal of harmful metals and metalloids and have proved to be efficient sorptive materials. A range of methods has been used to obtain them, but the coprecipitation method prevails in the cited examples. The differences in the structure and morphology of the obtained Fe_3_O_4_/biochar materials are presented in Figure 5. The magnetic properties that biochar acquires after it is combined with Fe_3_O_4_ significantly facilitate the process of removing pollutants from water and sewage by eliminating the problem of secondary contamination of watercourses with adsorbent residues. Moreover, the presence of magnetite in the structure of the carbonaceous adsorbent has a beneficial effect on the sorption of harmful metal ions, allowing, among others, their electrostatic interaction and surface complexation.

## 6 Materials with Biochar and Other Oxides

Over the past few years, TiO_2_, ZnO, and iron oxides have not been the only oxides combined with biochar. Along with the development of biochar applications, oxides of other metals were also investigated and details concerning the removal of impurities by composites based on biochar and those oxides collected through a literature study are presented in Table 4. Although there have been more successful attempts of metal modification of the surface of biochar, during this literature study only articles regarding materials in which the presence of metal oxide in the samples obtained was confirmed were taken into consideration.

**Table 4:** Data of Wastewater Treatment Processes Using Inorganic Oxide/Biochar Systems

material	feedstock	pyrolysis temp (°C)	surface area (m^2^/g)	pollution	initial pollution concentration (mg/dm^3^)	applied dose (g/dm^3^)	adsorption capacity (mg/g)	degradation method	removal eficiency (%)	ref.
ZrO_2_/biochar	wheat husks and paper sludge		29.621	Reactive Yellow 39	20	1.5		sonocatalysis	96.8	(120)
CeO_2_/biochar	paper waste and wheat straw	500	59.0	Reactive Red 84	10	1.0		sonocatalysis	98.5	(121)
V_2_O_5_/g-C_3_N_4_/biochar	rice straw	450		Rhodamine B		10.0		photocatalysis	99.7	(122)
Al_2_O_3_/biochar	chitosan	600		fluoride	20	0.1	196.1	adsorption		(123)
Al_2_O_3_-Fe_2_O_3_-FeOOH- Fe^2+^/biochar	commercial biochar			NO_3_–N	8.66	10.0	34.2	adsorption	∼70	(124)
					34.65				∼60	

As can be seen from Table 4, metal oxide/biochar systems were used in catalysis processes and adsorptions for pollution degradation such as organic dyes, fluoride, and nitrates. Khataee’s group, in addition to their previously described works, focused on the sonocatalytic properties of ZrO_2_/biochar and CeO_2_/biochar in organic dye degradation. A ZrO_2_/biochar nanocomposite was prepared by a modified sonochemical/sol–gel method and applied as a catalyst in sonocatalytic degradation of Reactive Yellow 39.^120^ High sonocatalytic activity can be caused by the mechanisms of sonoluminescence and hot spots. The dye degradation efficiency was increased by increasing the ZrO_2_/biochar dosage and ultrasonic power and decreasing the natural solution and initial dye concentration. In the case of CeO_2_/biochar a hydrothermal synthesis method was applied and the obtained material was used in sonocatalytic degradation tests of an organic dye—Reactive Red 84.^121^ The catalyst efficiency was enhanced with the increase of catalyst amount and ultrasonic power but diminished with the increment in dye concentration and pH value. Researchers suggested that the percentage of OH radicals plays the key role in the process on the basis of the presence of quenching effects of various scavengers. Another organic dye subjected to photocatalysis degradation of metal oxide/biochar composite was Rhodamine B. Zang et al.^122^ synthesized biochar/vanadium pentoxide/graphite-like carbon nitride (biochar/V_2_O_5_/g-C_3_N_4_) using a simple hydrothermal method and subjected it to photocatalytic degradation of Rhodamine B (RB) under simulated solar irradiation. The hybrid material demonstrated a highly improved photocatalytic activity in comparison to its pristine components, reaching an RB adsorption capacity of 196.1 mg/g. So far, this has been the only mention found about the combination of vanadium oxide compounds with biochar and application to water purification. Considering the high catalytic performance of V_2_O_5_, it seems to be a correct direction to conduct research and this field should be given more attention in the future.

Research on the removal of inorganic pollutants from water was also carried out. Jiang et al.^123^ prepared Al_2_O_3_/biochar by employing chitosan (CS), poly(vinyl alcohol) (PVA), and AlCl_3_·6H_2_O as the raw materials, and used it as a fluoride adsorbent. The adsorption process was mainly governed by a chemical reaction, including ion sharing and transferring. The maximum adsorption capacity of fluoride reached 196.1 mg/g, this being a satisfactory result. You et al.^124^ used iron and aluminum oxide modified biochar for nitrates removal. A coconut shell biochar was modified by a solution of a mixture of FeCl_3_ and AlCl_3_, and after that composition studies revealed that iron and aluminum elements existed on the surface of Fe–Al/biochar in the form of FeOOH, Fe_2_O_3_, Fe^2+^, and Al_2_O_3_ respectively. Nitrates adsorption onto such adsorbents was endothermic and spontaneous as well as favored in an acidic condition. The maximum adsorption capacity of the tested material fitted by the Langmuir model could reach 34.20 mg/g. Ligand exchange and a chemical redox reaction were found to be the responsible mechanism in that process.

Despite several successful attempts to synthesize materials based on metal oxides and biochar, there is still much more to investigate in the field of their synthesis and application. The future of such materials appears to bright and they have a great deal to offer in the field of environmental protection, especially in water purification.

## 7 Possible Degradation Mechanisms

**Figure 6:**
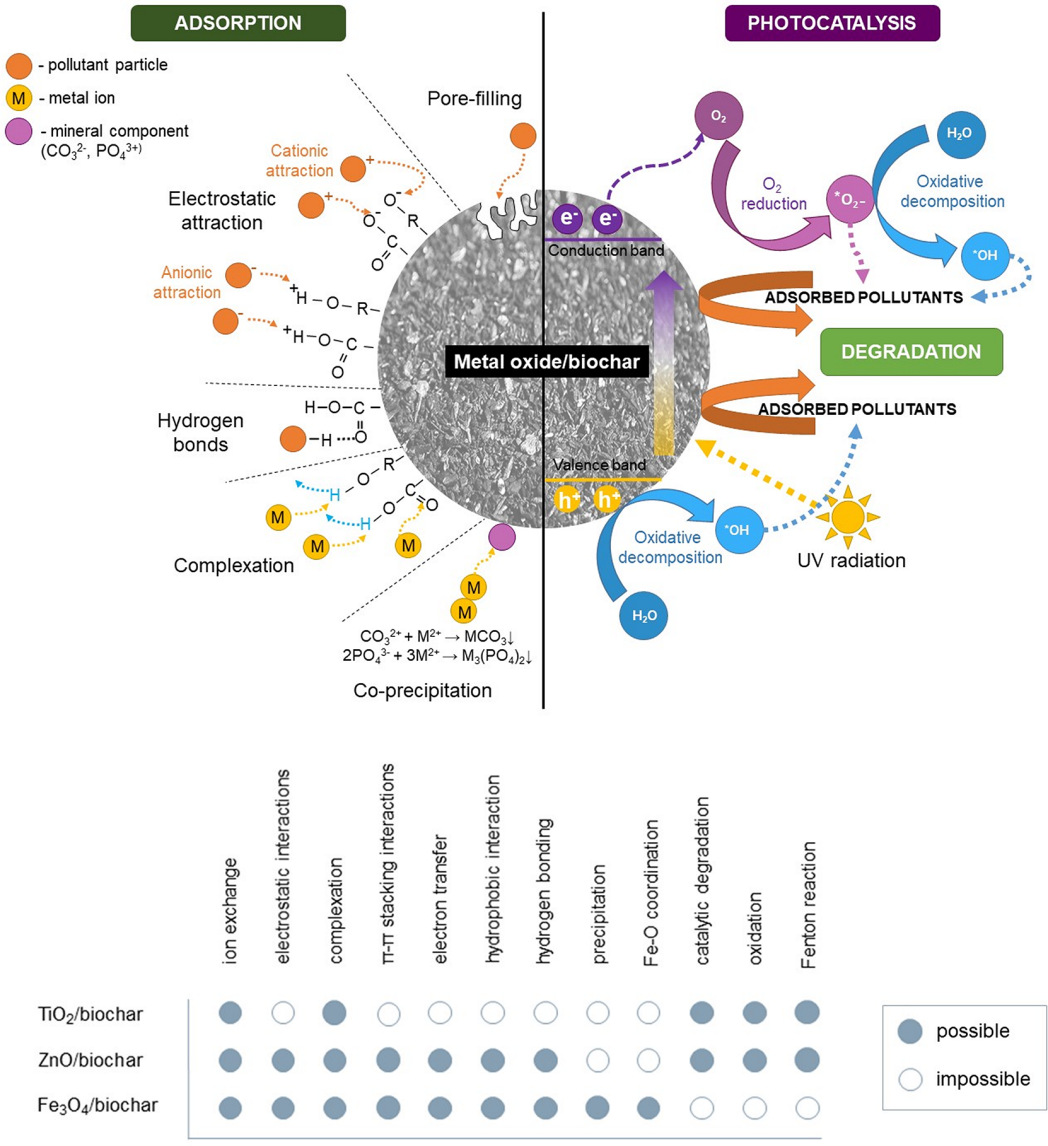
Schematic representation of main degradation mechanisms appearing onto metal oxide/biochar materials.

In the case of the combination of titanium dioxide and biochar, highly porous materials revealing an improvement in sorption and photocatalytic properties in relation to both pure TiO_2_ and biochar are obtained. The improvement of sorption properties is connected with an increase in the specific surface and porosity of those hybrids, while improved catalytic performance is related to the higher charge separation, caused by decrease of the energy gap and the reduction of electron–hole pair recombination. The incorporation of biochar into TiO_2_ enabled photocatalysis in visible light—a phenomenon that is impossible for pristine titanium dioxide. The removal of pollutants by TiO_2_/biochar hybrids took place mostly by catalytic degradation, oxidation, and Fenton processes. In the majority of cases, the introduction of zinc oxide into the structure of biochar improved the morphological properties and the porous structure of the obtained hybrids, which translates into an improvement in the sorption properties of various pollutants. Such modification enhances the positive surface charge of the hybrid material, increasing its affinity to anionic species. Biochar-derived functional groups on the surface of a ZnO/biochar material increase its sorption properties but do not affect its photocatalytic activity. Degradation of pollutants using ZnO/biochar occurs through both adsorption and photocatalysis, according to mechanisms such as ion exchange, electrostatic interactions, π–π stacking interactions, electron transfer, inner-sphere complexation, oxidation, and Fenton processes. The main motivation for modifying the biochar with Fe_3_O_4_ was to give the hybrid material magnetic properties in order to facilitate its separation. Furthermore, it appeared that the addition of magnetite has a positive effect on the sorption properties, increasing the surface area of the adsorbent and enabling the adsorption by Fe–O coordination. The mechanism of adsorption of pollutants on Fe_3_O_4_/biochar hybrid materials was explained by conjugation adsorption, diffusion, precipitation, ion exchange, surface complexation, electrostatic interactions, hydrogen bonding, and hydrophobic interactions. The main mechanisms of pollutant degradation by metal oxide/biochar materials are shown schematically in Figure 6.

## 8 Conclusions

This paper has dealt with the removal of pollutants from water and wastewater with the use of materials based on biochar and three inorganic oxides TiO_2_, ZnO, and Fe_3_O_4_. The hybrid materials were obtained by a multitude of methods, including pretreatment processes such as impregnation of biomass with metal salts, coprecipitation, sol–gel, and solvothermal methods, as well as post-treatment processes such as biochar impregnation, direct hydrolysis, ball-milling and mechanical mixing of components. All of these methods enabled the efficient creation of the inorganic oxide/biochar hybrid material, which was confirmed by the results of physicochemical analyses. The combination of inorganic oxides with the biochar results in a visible improvement of the existing properties or gives new properties to the hybrid materials, desired in the wastewater purification processes. Biochar, as an practical waste material, in combination with excellent photocatalysts such as TiO_2_ and ZnO or magnetic Fe_3_O_4_, can reach a reasonable position in the removal of various impurities in processes of adsorption and photocatalysis. All of the discussed materials showed satisfactory efficiencies in the elimination of organic pollutants, pharmaceuticals, and harmful metal ions. In the preceding section the most probable mechanisms of degradation of environmental pollutants by metal oxide–biochar hybrids were discussed. The review presented in this paper will probably not exhaust the potential applications of TiO_2_-, ZnO-, and Fe_3_O_4_/biochar materials, and there is still a great deal to be investigated in this field.
